# Towards the effects of translators’ emotional intelligence and anxiety on their translation quality

**DOI:** 10.1016/j.heliyon.2023.e19276

**Published:** 2023-08-19

**Authors:** Guanghua Qiu

**Affiliations:** Lecturer in English Curriculum and Teaching Theory and Translation, School of College English Teaching and Research, Henan University, No.85, Minglun Street, Shunhe District, Kaifeng, 475000 10260036, People's Republic of China

**Keywords:** Translation quality, Anxiety, Emotional intelligence, Translator

## Abstract

For years, the impact of translators and their emotional characteristics on translation quality have been the main concern of scholars. That is, few studies have evaluated the role of translators' qualities on their professional performance. It is because most translation studies have focused on the linguistic and sociolinguistic determinants of translation quality. To highlight the consequences of translators' emotional characteristics on their professional performance, this study sought to review the role of translators’ emotional intelligence and anxiety on their translation quality. As evidenced by previous investigations, both emotional intelligence and anxiety can enormously affect the quality of translations. This review offers some takeaways for educators in translation studies as well as the translation educational system to make them cognizant of the role of emotional intelligence in translation quality. It also recommends some solutions to avoid the adverse effects of anxiety on translation quality.

## 1 Introduction

In the era of global communication and cultural discourse, translation plays a vital role in transferring different ideas among nations. House [1] believed that translation provides access to existing messages, but it is a secondary form of communication. The translation is considered a challenging and problematic area, and it is an issue that significantly influences daily life [2]. A piece of the translated text reproduced on a sheet of paper is not a simple translation; many things are involved in the process of translating a text; not all of which the translators are aware. Most importantly, some features are involved that are not visible to the naked eye, and these are the features that are taking place in the translators' minds while translating a text [3]. Hansen [4] suggested that translators’ decision procedures are influenced by cognitive and emotional experiences. He asserted that the whole process of translation occurs in the mind of the translator. So, to translate a text, the translator focuses on reading, thinking, analyzing, and comprehending the target transcript.

Translators have a crucial role in translation, and many factors will affect the quality of their translations and psychology is one of these disciplines and has a strong connection with translation. Mentally, feelings and having knowledge about them is a crucial aspect, and presumably it can greatly impact the translation tasks [5]. In other words, these psychological aspects have prominent impacts on the performance of translators, and they can affect translations or influence translators' decisions. One of the recent investigations in psychology is the exploration of emotional Intelligence which is regarded as one of the utmost significant aspects of intelligence that is one of the factors that appear to have a notable impact on influencing the mindset of translators during the translation process and thereby aiding in the translation task [6]. The emotional intelligence construct is defined by Salovey and Mayer [7] as “the ability to monitor one's own and other's feelings and emotions, to discriminate among them and to use this information to guide one's thinking and actions” [p. 189]. Higher intelligent translators, in terms of emotions, can better moderate and control such external and internal factors. Furthermore, Ravakhah, Dastjerdi, and Ravakhah [8] noted that “translation is a mental process needing sufficient concentration. Anxiety is a mental disorder that makes the mind busy and can lead to lack of concentration or decentralization, and anxious people cannot do their tasks” [p. 1053]. A heavy workload, the complexity of translating texts, short time constraints for translation, and unfamiliarity with the texts being translated are some of the factors that contribute to the translators' anxiety. When feeling anxious, translators are unable to concentrate on the text and are unable to produce a high-quality translation due to the lack of comprehension of the texts to be translated [9]. As stress triggers decentralization, anxious translators would struggle to focus on the original text and produce a high-quality translation because decentralization hinders their comprehension of the original material [8].

Anxiety needs to be explored since it can inhibit individual performance in cognitive task efforts [10,11]. The translators, with higher levels of anxiety, are not able to concentrate on the source and target texts, and they are also not able to produce an appropriate translation because anxiety sets a limit in getting the notion of the source texts [10]. Oraki and Tajvidi [12] asserted that with the expansion of communication and the need for the development of relationships, the need for (oral) translators, as an intermediary between the two sides who do not understand each other, has risen day by day. He mentioned that the most important condition for oral translation is the power of the translator to communicate effectively and to transfer accurately and reliably. In written translation, there is a time for reviewing, referring to dictionaries, and correcting, while oral translation is a demanding task requiring a high level of attention and presence of mind, and is something like magic in the brain. Oral translators in this sophisticated, fast, and intensive process of data exchange, in addition to full coverage of both the source language and target language and subject matter of the speech, must also have a high speed. A person who intends to enter the profession of oral translation must focus on his/her memory and body language. S/he must also be aware of the challenges faced during translation, including time, speed, concentration, accuracy, stress, anxiety, sensitivity to responsibility, and ways to present a decent oral translation. Therefore the consideration of anxiety in translation performance is critical for translators [12].

This review is included in translation psychology. According to Núñez and Bolaños-Medina [13], translation psychology is “an emerging area of translation studies” [p. 1]. According to Hansen, Chesterman, and Gerzymisch-Arbogast [14], translation psychology covers “research which focuses primarily and explicitly on the agents involved in the translation, for instance on their activities or attitudes, emotions, their performance and their interaction with their social and technical environment, or their history and influence” [p. 13]. Another underpinning field in this review is Positive Psychology (PP). It aims at identifying human characteristics and the way they create psycho-emotional resources to flourish in their life [15,16]. PP is tentatively supported by the broaden-and-build theory. In other words, being subjected to emotional states, including happiness, love, satisfaction, involvement, resilience, grittiness, and attentiveness, “broaden people's momentary thought-action repertoires and build their enduring personal resources” [17]. p. 2]. PP, as a new perspective on foreign language education, has been developed over years [e.g., 16,[18], [19], [20]. Emotional intelligence, as a key term for emotion regulation, is a relevant notion to PP.

This review strives for probing the investigations on the effect of translators' emotional intelligence and anxiety on translators' performance in translation. Given that Emotional Intelligence affects academic success [21], and comprehension of different viewpoints [22]), the significance of examining its influence on the quality of translations is emphasized even more. Additionally, there has been thorough research regarding the significance of anxiety related to foreign languages, as well as anxiety related to one's current emotional state and long-standing personality traits, in terms of academic achievement [10,17,23]). Nevertheless, the investigator in this analysis endeavors to explore the significance of anxiety encountered by translators and investigate the correlation between their EI, anxiety, and the quality of their translation.

As a result, this review can be important for translation students since they can be aware of emotional intelligence, anxiety, and their effects on translation skills. Translation trainees can benefit from this study because they will have a greater view of students’ emotions, and consequently they can help learners develop their knowledge by training their metacognitive strategies. Also, one of the most productive ways to support students by understanding emotional intelligence and anxiety is by helping learners develop their self-regulation – the aptitude to control opinions and emotions.

## 2 Literature review

### 2.1 Translation and emotions

Newmark [24] pointed out that translation refers to “rendering the meaning of source text into the target language in the way that the writer intended the text” [p. 133]. House [1] indicated that translation is a linguistic act in communication across cultures. Hodgson [25] mentioned that translation should not be considered a one-dimensional topic, but it should be regarded as “a composite, interdisciplinary network of data, methods, theories, and hypotheses from a vast majority of fields” [p. 140]. [26], p. 1082] also defined translation as “a cluster concept with an open definition”. He argued that the flexible nature of translation, along with a lack of exact limitations, has freed it to be familiarized with numerous cultural situations, social functions, and emerging technologies.

While the general theories and practices of translation have been widely studied, the psychological issues of translators have not been given appropriate attention [27]. During the 1990s, the emergence of ‘translation psychology’ sheds light on translators' traits, rather than the translation process [28]. Overall, emotion or feeling has been specified as a notable aspect of translators' information processing in translation, which is affected by various negative and positive emotional constructs [29]. The creativeness and quality of the translated text have been influenced by translators' feelings. Therefore, positive feelings increase the attention span and develop flexible and creative thinking, while negative emotional states decrease attention span and foster critical thinking and particular problem-solving manners [30]. Jääskeläinen [28] stated that the emergence of translation psychology “implied rethinking translation as a complex process involving human psychological factors succinctly addressed in the translation competence models” [p. 193]. Investigations on the psychological traits of translators underscore numerous facets encompassing the human nature of translators, such as the influence of their viewpoints [31], individualized characteristics [32], resilience [27], and positive and negative feelings [33] on the translation procedure.

Rojo and Caro [34] also investigated the effect of positive and negative feelings on the translation process, and their results indicated that different positive and negative emotional states induce different processing styles in translation as a cognitive process. They also argued that positive emotional states result in translators' more creative thinking, while negative emotional states lead to precise and regular processing. This review tries to brood over emotional intelligence, as a type of personality trait and anxiety to consider their roles in regulating translators’ performance in generating efficient translation.

### 2.2 The concept of emotional intelligence

Some definitions have been provided for the notion of emotional intelligence. O'Connor et al. [35] classified it into trait and ability. Petrides, Pita, and Kokkinaki [36] defined ability emotional intelligence as “the emotions which are related to cognitive-emotional abilities of individuals, requiring maximum performance measures” [p. 11]. In this regard, Schutte et al. [37] pointed out that emotional intelligence copes with the individuals' competence to differentiate, comprehend, control, and direct feelings. On the other hand, according to Ebrahimi, Khoshsima, Zare-Behtash, and Heydarnejad [38] emotional intelligence refers to “a trait in individuals which empowers them to regulate their feelings and the emotional states of others, distinguish between diverse feelings, and apply emotional information to lead their thoughts and performances” [p. 627]. However, Salovey and Mayer [7] pointed out that “emotional intelligence is the ability to perceive emotions, to access and generate emotions to assist thought, to understand emotions and emotional knowledge, and to reflectively regulate emotions to promote emotional and intellectual growth” [p. 187].

In general, Bar-On [39] described emotional intelligence as “a cross-section of interrelated emotional and social competencies, skills and facilitators that determine how effectively we understand and express ourselves, understand others and relate to them, and cope with daily demands” [p. 3]. Many investigators distinguished between two emotional intelligence constructs, depending on whether the operationalization process is based on self-report (as in personality questionnaires) or maximum performance as in IQ tests [36]. Bar-On [40] elucidated that emotional intelligence is regarded as the series of non-cognitive aptitudes, and capabilities that affect a person's level of flexibility to the stresses and difficulties of the surroundings. He also divided emotional intelligence into “intrapersonal (emotional self-awareness, assertiveness, self-esteem, self-actualization, and independence), interpersonal (empathy, interpersonal relationships, and social responsibility), adaptability (problem-solving, reality testing, and flexibility), stress management (stress tolerance, impulse control), and general mood (happiness and optimism)” [p. 14]. Furthermore, Saud [41] integrated a few personality traits, such as self-possession, scrupulousness, and enthusiasm for accomplishment into the definition. Numerous studies have been conducted on learners' emotional intelligence and its association with their cognitive abilities, such as academic achievement [42], and language learning strategies. Çoban, & Telci [43] investigated the role of emotional intelligence of translators and interpreters. Emotional intelligence includes skills like personal, emotional, and social capabilities. Their review indicated that universities providing translation courses are not prepared with the sub-components of emotional intelligence such as self-awareness, compassion, communication skills, and the capability of identifying and transmitting own and other individual's feelings.

### 2.3 The role of emotional intelligence in translation performance

It is stated that translation is a type of cognitive activity that requires resourceful problem-solving skills in new, contextual, communal, and cultural circumstances. Emotional Intelligence is considered one of the primary issues that controls translators' mindsets and help them be innovative in translation activities [38]. Given the individualized differences in emotional intelligence scores, in the review study by Hubscher-Davidson's [44], it was revealed that there was a significant difference between literary and non-literary translators. Her investigation showed that translators with greater emotional intelligence are more inclined to control their feelings and organize the emotional nature of source and target texts. She argued that “being able to appraise and communicate one's own and other people's emotions is a key aspect of intercultural communication, and therefore a key skill for translators and interpreters” [p.332]. She mentioned that translators with higher levels of capability to appreciate individuals' feelings are more likely to be more efficacious in opting for proper strategies for rendering the source language into the target language. She also asserted that translators' emotional intelligence is critically important in understanding the processes of translation. She argued that emotional intelligence affects the quality of translation utilizing perception, sympathy, and creativity. She pointed out that “if there are certain emotional and personality-related attributes (e.g., intuition, sensitivity, and empathy) that successful creative writers have been found to share, there may also be certain attributes that successful literary translators may also share” [p. 334]. Hubscher-Davidson [44] contends that translation, as a technical skill, relies on the creativity of the translator in conveying the intended message into the target language. She also underscored the need to incorporate emotional intelligence into translation studies. She focused on the requirement of integrating emotional intelligence into translation research and understood a connection between special personality traits and translation quality by using Myers-Briggs Type Indicator (MBTI) test extensively utilized in psychology to make character analysis.

In line with Hubscher-Davidson [44], Pahlavani and Asroush [45] examined if there is a correlation between the level of EI in Iranian EFL learners and their proficiency in English-Persian oral translation. In order to achieve this, seventy-two out of ninety university students majoring in English translation were selected after ensuring their similar abilities. Afterward, all participants were provided with guidance and strategies for oral translation by the teacher and completed identical assignments. Finally, a final oral translation examination and an EI scale were conducted for all participants. The result showed that translators' emotional intelligence, is considered a significant component in manipulating their minds. Their study showed that there is a significant correlation between the interpreters’ emotional intelligence and their attainment in translation skills.

Varzande and Jadidi [46] underscored the significance of translators' emotional intelligence. A representative sample of one hundred male and female expert translators were chosen utilizing purposive sampling technique and they were requested to fill out the Trait Emotional scale. The respondents were subsequently instructed to convert a passage consisting of 232 words, from Orwell's 1984 book, in order to evaluate the quality of their translation. They argued that an emotionally intelligent translator is an adjusted expert who inspires confidence, and this partly shows the level of achievement in his or her occupation. They proposed the integration of emotional intelligence in translators' training courses. Moreover, Ebrahimi, Ahmadnejadian, and Zarafshan [38] investigated translators' emotional Intelligence and their translation accuracy. To this end, 41 students fillied out the emotional quotient scale and they used political texts as translation manuscripts, in which emotions play leading roles. Using EQ-I questionnaires, they decided to estimate the emotional intelligence of the translators who are required to translate a text in different stress-free environments and stressful situations. They also scrutinized the translation of each translator meticulously. The analysis of the data revealed that translators with higher emotional intelligence were inclined to control their emotional states, and their accuracy of translation was high since they tried to select the closest equivalent for the word. In contrast, translators with lower levels of emotional intelligence would translate more conservatively to avoid any possible unwanted consequences. They also argued that different texts bring about different emotional involvement among translators.

Besides, Shadman, Khoshsaligheh, and Pishghadam [47] used Petrides' [48] facets of emotional intelligence to study the influence of emotional intelligence on translators' performance in translation. The research examined a purposive selection of published interviews with 16 renowned modern Iranian language literary translators. The factors include well-being, self-control, emotionality and sociability. They found that among well-being, self-discipline, emotionality, and sociability as the indicators of emotional intelligence, self-esteem (as a sub-category of well-being), and self-motivation (as a sub-category of sociability) are more significantly correlated with translators' translation performance. Also, Tajvidi and Ferdowsi [49] investigated the association between the translators' emotional intelligence, gender, and their translation quality in simultaneous interpretation. In order to achieve this goal, a total of 70 English Translation students enrolled in the Oral Interpretation course were chosen. The participants were administered the Emotional Quotient Inventory scale and were additionally requested to complete an oral cloze exam. They found a significant correlation between tolerance and flexibility, as the sub-components of emotional intelligence with the translators’ performance in translation.

Ghobadi, Khosroshahi, and Giveh [50] examined the contribution of translators' emotional intelligence, ambiguity tolerance, and working memory, to translators' performance in translation. To conduct the research, a total of fifty-four Iranian postgraduate students studying Translation were chosen as subjects and they filled out the relevant questionnaires. Furthermore, they were asked to translate a passage from English to Persian. The results of multiple regression analysis revealed that these variables interacted with each other to predict the quality of translation. They argued that translation is a representational task, and therefore, it is assumed that translation is significantly influenced by the translators' mental developments and their willingness to use these processes in doing translation tasks. They asserted that translators with higher levels of emotional intelligence are widely responsive to linguistic and non-linguistic relations, and they can produce high-quality translations. Ghaemi and Bayati [51], using structural equitation modeling, investigated the association between 34 qualified translators’ emotional Intelligence, burnout, and translation skill. Their study revealed a significant correlation between emotional intelligence and translation skill. They justified their study by arguing that the translators with higher levels of emotional intelligence, social, flexibility, and stress management competence, tend to produce a high-quality translation. Cheng [52] explored the impact of critical thinking and emotional control on the performance of translation students. The literature has already established a strong and meaningful connection between the critical thinking abilities of translation students and their performance. Additionally, research has indicated that the regulation of emotions and related factors like resilience, EI, and professional expertise can have a significant impact on translation performance.

### 2.4 The concept of anxiety

According to Králová [53], “anxiety refers to the negative vibes and feelings that cannot be seen or controlled by students i.e., it is associated with the feeling of nervousness, fear, threat, frustration, helplessness, and hopelessness” [p. 3]. It is considered as a psychological sign of stress. Anxiety can be categorized into a trait, state, and situation-specific anxiety [54]. Ellis [55] stated that state anxiety refers to “the apprehension that is experienced at a particular moment in time as a response to a definite situation” [p.145]. In the same vein, Alshahrani [56] also asserted that state anxiety is regarded as a temporary emotional state that individuals may experience in the time of adversary. Elwood et al. [57] pointed out that “trait anxiety is an individual's tendency to appraise situations as threatening, avoid anxiety-provoking situations, and demonstrate high baseline physiological arousal” [p. 23]. Likewise, Erdiana et al. [58] specified that trait anxiety has stability as its component and it made individuals feel uneasy in different contexts. Situation-specific anxiety, on the other hand, is triggered by a specific condition, say, public speech or class involvement [55].

Horwitz, Horwitz, and Cope [59] indicated that foreign language anxiety is a subcategory of situation-specific anxiety. In another classification, Pascoe et al. [60] placed foreign language anxiety in the main category of negative emotions, such as educational-based anxiety, which has an emotional impact on emotional and physical well-being. In an instructive environment, Horwitz, Horwitz, and Cope [59] pointed out that foreign language anxiety is “a distinct complex of self-perceptions, feelings, and behaviors related to classroom language learning arising from the uniqueness of the language learning process” [p. 127]. Gardner and MacIntyre [61] stated that “foreign language anxiety is fear or apprehension occurring when a learner is expected to perform in the second or foreign language” [p. 59]. Numerous investigators [e.g., [62], [63], [64], [65] have underlined foreign language anxiety in the educational scope.

Horwitz, Horwitz, and Cope [59] characterized the construct of foreign language anxiety into “a) test anxiety, b) fear of negative evaluation, and c) communication apprehension” [p. 30]. Cakici [66] declared that test anxiety is considered a student's fear of suffering disappointment in educational assessment in testing environments. He also specified that negative evaluation is related to the concern about persons' negative decisions. Communication apprehension, based on Horwitz, Horwitz, and Cope's [59] definition, refers to “a distinct complex of self-perceptions, beliefs, feelings, and behavior related to classroom language learning arising from the uniqueness of the language-learning process” [p. 28]. Communication apprehension is an important component in restraining the received comprehensible input, and it affects accomplishments in instructive environments [67].

### 2.5 The role of anxiety in translation performance

Some investigations have been conducted on the association between anxiety and cognitive skills [e.g., [68], [69], [70]. Translation is regarded as one of the cognitive skills, which is affected by anxiety [e.g., 32,70,71. Ravakhah et al. [8] studied the influence of anxiety on translators' performance. They used Beck and Steer's Anxiety Inventory to collect information about translators' anxiety. Their results showed the insignificant effect of anxiety on translation accuracy. However, they found that translators' translation speeds vary with various anxiety levels. In other words, their study revealed that the lower-level anxious translators rendered the texts in a shorter time than the anticipated time. They argued that nervous translators focus on the source text laboriously and therefore, perform in a longer time than other translators. They maintained that “the minds of anxious translators are too busy; they think about their problems and search for solutions” [p. 5].

Pezeshki [72], in his study, examined the impact of anxiety on translators' oral translation through Dundee's Stress State Condition Questionnaire. This questionnaire can provide valuable information about how much an individual's situation changes under pressure, and this questionnaire is concerned with the feelings and thoughts of the person during the task. He also employed an oral translation test, which was developed to assess each translator's capabilities by translating three distinct audio files. Translation trainees, in the first phase of the test, were asked to listen to three audio files sequentially with 5 s to play the next audio file and take notes. In the second phase of the test, instead of oral translation, they were asked to write what they understood in 10 min on paper so that evaluators can evaluate translation trainees' responses. He found a negative and significant correlation between translation trainees' anxiety and their performance in oral translation. He justified his result by the time length of the translation exam. He mentioned that short time length can cause anxiety among translation trainees. Anxiety in the process of translation pertains to the apprehension and conduct of translators during the conversion of two languages. It is one of the significant elements impacting the emotional alteration of translators [73]. During the process of translating from Chinese to English, translators may experience psychological anxiety caused by certain translation challenges. This underlying state of unhealthiness can greatly hinder translation work and have significant negative impacts on the translators' physical and mental well-being [74].

Skolastika [75] investigated the reasons for anxiety in oral translation, and he offered some translation strategies to deal with this type of negative emotion. His study showed that anxiety mainly occurs when translation students realize the key meaning of the source language but are not able to render it into the target language. Moreover, he found that translation students tend to be more anxious before beginning their oral translation. He attributed his results to some issues, such as fear of stumbling, deficiency in practice, time restrictions, and inadequacy in self-confidence. Rojo López et al. [34] underscored that translation performance is affected by time pressure. They measured three consequences of time pressure, including salivary cortisol, self-esteem, and anxiety. Thirty-three trainees in Spanish translation translated 3 literary texts in English under varying time constraints: Text 1 (unlimited time), 10 min for text 2, and 5 min for the last text. Their study demonstrated that translators' higher state of anxiety is significantly correlated with the employment of a decreased amount of rendered words. However, they also found out that higher trait anxiety causes higher accuracy under time pressure and the absence of time pressure. They argued that overloading working memory under time-pressure tasks is correlated with higher levels of trait anxiety and it impairs translation speed. Writing anxiety, as another source of anxiety, affects translation performance. Yan et al. [71] explored the role of translation students' anxiety concerning writing skill and their performance in translation. The individuals involved in this research were fifty pupils specializing in translation at a higher education institution in Hong Kong. They were asked to fill out the students' anxiety scale when writing English and the results of the study indicated that foreign language writing anxiety negatively affected translation performance. They proposed some approaches to foster translation students’ foreign language writing competence in the translation curriculum.

Lopez and Naranjo [30] studied the emotional effect of contextually-based source texts and their Spanish translation students' performance in translation and reading comprehension. 69 Spanish translation undergraduate pupils offered to participate in the study. For the purpose of the study, they were haphazardly assigned into two groups: the optimistic group and pessimistic group. Their study showed that translation learners’ anxiety levels in reading comprehension are significantly higher than their anxiety levels in translating. They argued that when attentional demands are high, task performance takes precedence over anxiety-related cognitive processing. Therefore, in reading comprehension translation, students typically focus on comprehension, and their emotional response tends to be influenced by negative emotional states. They mentioned that when student translators are absorbed in the translation activities which are demanding that engage decision-making resources, their levels of anxiousness will be decreased.

Rajabi and Yousefi [76] examined the association between anxiety levels and emotional intelligence of translation students during the performance of oral translation, and they also investigated the effectiveness of anxiety and emotional intelligence on translation performance. With the aim of achieving this objective, a group of 30 Iranian undergraduate students studying English translation were selected through convenience sampling to take part in this research. Subsequently, the participants were requested to sequentially translate a 3-min video clip on anxiety disorder in English to Persian. They employed Horwitz and Cope's [59] foreign language classroom anxiety scale (FLCAS), and Schering's emotional intelligence questionnaires to measure translation students' anxiety and emotional intelligence, respectively. Their study indicated that negative emotions are significantly correlated with emotional intelligence. Their findings also showed that anxiety and stress lessen students' capabilities in translation performance. They argued that self-regulated translation students have minor levels of anxiety in translation performance.

## 3 Conclusions, implications, and suggestions for further research

This review explored the role of translators' emotional intelligence on their translation performance based on the review of the literature; it was revealed that emotional intelligence has a key function in translation performance. In other words, most of the studies have shown that emotionally intelligent translators outperform their translation performance. Also, the association between translators' anxiety and their performance in translation was scrutinized. This review showed that translators’ anxiety negatively influenced written and oral translation. It is exemplified that negative emotions and feelings like stress and anxiety, decrease the eminence of learners' translation ability. As a result, the translation theoretical guidelines, along with teaching emotional issues should be deemed to train talented and expert translators. In order to establish a calm and professional classroom atmosphere, educators should empower students to manage their adverse emotions and mental state like stress and anxiety. This could be facilitated by teaching psychological strategies and techniques that aid in anxiety reduction. Furthermore, it is recommended that translation teachers participate in a psychological evaluation, like an anxiety assessment, early on in their teaching program to ascertain their students' feelings. This will enable them to formulate an effective and tailored study agenda for their students.

Moreover, earlier studies showed that a sufficient amount of stress has a positive impact on individual performance. Regarding the key role of psychology in translation, the current study suggests pedagogical implications for syllabus designers to supply some beneficial emotional courses to the translation program. Consequently, instructors should inspire trainees to control their destructive emotions and beliefs, like trauma and nervousness, and they should provide some emotional approaches and practices to lessen translators' apprehension and encourage a professional atmosphere. Furthermore, the research also indicates that translation teachers have the option to undergo a psychological examination, like an anxiety assessment, at the start of the translation program in order to identify their students' feelings. This will enable them to develop an appropriate and effective learning strategy for their students. Some implications have been provided for translator trainees and the translation educational system. To enhance the translators’ performance and promote the quality of translation, translator trainees must know how to deal with anxiety. If the translator trainers teach the translation students in their classes how to deal with and manage stressful situations, translators will control these situations as well, and they will not lose their concentration. So, the translators will be able to avoid the negative effects of anxiety and high stress in those conditions. Translator instructors should be conscious of the personalities of their students, and they should acknowledge the requirements of translation learners and assist them in discovering their own resolutions to translation difficulties. Emotional intelligence, as a constituent of individual characteristics, is seen as a controller of translators' feelings. The trainers of translators must offer motivating and pleasurable translation assignments that stimulate their EI and alleviate linguistic anxiety in their thoughts. Therefore, the inclusion of pleasurable assignments can control translation students' emotional intelligence to enhance their translation proficiency. These concerns decrease students' mental burden and cultivate their translation excellence.

Based on the review of the literature, it can be concluded that translation educators should not disregard the vital power of their student translators' emotional intelligence in the process of translation as translation is not only a problem-solving but also a decision-making route that is prejudiced by emotional intelligence. It is also recommended that the translation scholastic system develops a widespread syllabus for the advantage of all student translators respecting their emotional intelligence. To develop translators' emotional intelligence, it is required to boost the sub-categories of emotional intelligence by providing activities and tasks that employ a blend of evidence-based approaches and procedures. Moreover, operative questioning can be a key teaching tool that can assist translators to comprehend and recognize their feelings. Various kinds of questions are probably suitable according to numerous issues. For example, closed questions can be suitable for starting a conversation, but open questions are typically recognized to be beneficial in training conversations, whereas leading questions are regarded as controlling questions and are not acclaimed. Translator trainees should highlight standards and individual powers because of their potential for increasing awareness of positive individual characteristics that might have been extensively overlooked or undervalued. Standards are guiding indicators that can assist individuals to systematize their individual and professional lives. Standards are important components in human motivation and assist individuals they make up their minds. A person-centered approach should be adopted in training contexts. The strengths-based exercise is an activity that allows translators to reflect on the values. Translator trainees can provide an exercise in which translators are required to recognize and evaluate their values. Translator trainees should also use lectures, role-plays, group discussions, readings, and personal diaries to develop translators’ emotional intelligence. Furthermore, the various sorts of translations require particular skills in the discernment, processing, control, and operation of affective information which are exclusive to each translation type.

This investigation also contributes to the literature related to stress that has central implications for both translator educators and students. For instance, translator educators can arrange tasks that improve translators' coping strategies to control their anxiety and nurture emotional issues. They should be conscious of themselves and identify the adaptive responses and approaches to alleviating their stress and anxiety in case of confronting stressful situations and they should be helped when they are supposed to run into their numerous requirements, containing emotional and spiritual ones. The new schedules have to be fixed by translator's trainers to train the translation students at universities how to cope with their high level of anxiety and organize it in a direction to help them increase their concentration and performance. So, during the semesters the professors or the translator trainers can create different stressful atmospheres in the classroom to make it usual for students to behave in stressful situations easily and with relaxation and with high concentration; accordingly, they must practice routinely. Translation students must also learn how to restrain their anxiety. Perhaps the best way to control anxiety is to calm down. So instead of fighting anxiety, you can relax. The most important step in reducing anxiety is to make it a cut. That is the belief that: “I have anxiety and it's not my own hand” gives its place to a new belief that “I am anxious and try to reduce it by using different methods”. With this new belief, the feeling of inability to control anxiety gradually gives its place to the feeling of an ability to control anxiety and this will have a positive effect on performance. Translator trainees can provide some opportunities for translation students to relieve their anxiety. Music therapy is an emerging approach that can be offered to translation students which enables learners to increase their emotional intelligence [77].

It is also suggested that the translation educational system cultivates an inclusive set of courses for the advantage of all learners regarding their levels of emotional intelligence. One would hope that this review will expose novel perspectives to translation learners and translation instructors. To increase student translators, the translation educational system can hold academic workshops to make changes in learners’ emotions. Translation educational systems should employ knowledgeable trainees since educational knowledge can be a significant matter for enhancing emotional intelligence and dropping anxiety among students. The translation educational system must boost instruction efficiency by decreasing foreign language anxiety among translators. It should also inspire training in schoolrooms, which inspires emotional intelligence. Drawing on the essential role of psychology in translation, the current study provides pedagogical suggestions for curriculum planners to incorporate beneficial psychological modules into the translation syllabus. The trainers of translators must provide translation tasks that stimulate emotional intelligence and diminish language apprehension, while also being engaging and inspiring. To enhance the translation skills of students, it is important to incorporate pleasurable activities that can effectively regulate their emotional intelligence.

Subsequent studies can explore the effect of other personal traits on the performance of translators. Future research could also focus on examining the relationship between personality traits and emotional intelligence with other creative strategies on a group of professional translators. Also, interested researchers can compare different measurement models of emotional intelligence to investigate the association between translators' emotional intelligence and their preference for using different translation strategies. The other variables, such as translators’ socioeconomic class, and emotional issues can be taken into consideration by further investigations. Furthermore, more inquiries need to be conducted to triangulate the results by utilizing multidimensional research methods [78]. Future research should intensely inspect the effects of life stressors on job evolution among the precise groups. In short, future studies can be concluded to explore across areas to realize if such stressors become worse in diverse geographical parts [79]. Also, the association between translation quality and positive psychological constructs can be examined in the upcoming research. The effect of other negative emotions, including boredom, disappointment, burnout, and anger on translation performance can be investigated [80]. Finally, the impacts of emotional intelligence on translation accuracy, and translation fluency can also be compared.

## Funding

This study is supported by Research Project on Teacher Education and Curriculum Reform of Henan Province in 2023 (A Research on the Cultivation of Cross-cultural Ability of Undergraduate English Majors in the Perspective of International Communication) under Grant number (2023-JSJYYB-128).

## Author contribution statement

All authors listed have significantly contributed to the development and the writing of this article.

## Data availability statement

No data was used for the research described in the article.

## Additional information

No additional information is available for this paper.

## Declaration of competing interest

The authors declare that they have no known competing financial interests or personal relationships that could have appeared to influence the work reported in this paper.
